# The Modulatory Properties of Li-Ru-Kang Treatment on Hyperplasia of Mammary Glands Using an Integrated Approach

**DOI:** 10.3389/fphar.2018.00651

**Published:** 2018-06-19

**Authors:** Shizhang Wei, Liqi Qian, Ming Niu, Honghong Liu, Yuxue Yang, Yingying Wang, Lu Zhang, Xuelin Zhou, Haotian Li, Ruilin Wang, Kun Li, Yanling Zhao

**Affiliations:** ^1^Department of Pharmacy, 302 Hospital of People’s Liberation Army, Beijing, China; ^2^Department of Traditional Chinese Medicine, First Affiliated Hospital of Chinese PLA General Hospital, Beijing, China; ^3^China Military Institute of Chinese Medicine, 302 Hospital of People’s Liberation Army, Beijing, China; ^4^Department of Integrative Medical Center, 302 Hospital of People’s Liberation Army, Beijing, China

**Keywords:** Li-Ru-Kang, hyperplasia of mammary glands, modulatory properties, metabolomics, network pharmacology

## Abstract

**Background:** Li-Ru-Kang (LRK) has been used in the treatment of hyperplasia of mammary glands (HMG) for several decades and can effectively improve clinical symptoms. This study aims to investigate the mechanism by which LRK intervenes in HMG based on an integrated approach that combines metabolomics and network pharmacology analyses.

**Methods**: The effects of LRK on HMG induced by estrogen-progesterone in rats were evaluated by analyzing the morphological and pathological characteristics of breast tissues. Moreover, UPLC-QTOF/MS was performed to explore specific metabolites potentially affecting the pathological process of HMG and the effects of LRK. Pathway analysis was conducted with a combination of metabolomics and network pharmacology analyses to illustrate the pathways and network of LRK-treated HMG.

**Results**: Li-Ru-Kang significantly improved the morphological and pathological characteristics of breast tissues. Metabolomics analyses showed that the therapeutic effect of LRK was mainly associated with the regulation of 10 metabolites, including *prostaglandin E2*, *phosphatidylcholine*, *leukotriene B4*, and *phosphatidylserine*. Pathway analysis indicated that the metabolites were related to arachidonic acid metabolism, glycerophospholipid metabolism and linoleic acid metabolism. Moreover, principal component analysis showed that the metabolites in the model group were clearly classified, whereas the metabolites in the LRK group were between those in the normal and model groups but closer to those in the normal group. This finding indicated that these metabolites may be responsible for the effects of LRK. The therapeutic effect of LRK on HMG was possibly related to the regulation of 10 specific metabolites. In addition, we further verified the expression of protein kinase C alpha (PKCα), a key target predicted by network pharmacology analysis, and showed that LRK could significantly improve the expression of PKCα.

**Conclusion**: Our study successfully explained the modulatory properties of LRK treatment on HMG using metabolomics and network pharmacology analyses. This systematic method can provide methodological support for further understanding the complex mechanism underlying HMG and possible traditional Chinese medicine (TCM) active ingredients for the treatment of HMG.

## Introduction

Hyperplasia of mammary glands is one of the most common breast diseases in middle-aged women and accounts for more than 70% of all breast disease (Chen et al., 2015). HMG is easily overlooked because of its clinical characteristics until the generation of mammary carcinoma. (Zhang et al., 2012). The morbidity of HMG has increased due to increased work-related stress and competitive career pressures along with the fast pace of modern life (Li et al., 2017a). However, there is still not a sufficient understanding of the etiology of HMG, and pertinent therapeutic strategies are limited. Hormone or endocrine therapy is one of the most commonly used methods to mitigate the clinical symptoms of HMG. Nevertheless, the side effects also decrease the quality of life for patients who receive long-term treatment. Surgical treatment as a form of therapy is hardly accepted by most patients with recurring symptoms (Henry, 2014). Therefore, finding a more appropriate treatment with fewer side effects and more therapeutic advantages is the current goal standard for treating HMG.

Traditional Chinese medicine (TCM) has been practiced in China for thousands of years to treat acute and chronic diseases. Its application in the prevention and treatment of HMG has garnered increasing attention. To explore the mechanism of action and active substances of TCM, a variety of constructive technologies have been used over the past few decades, such as metabolomics, proteomics, genomic arrays and network pharmacology (Xu et al., 2017). However, few researchers fully integrate the aforementioned techniques.

Li-Ru-Kang is composed of *Curcumae radix*, *Prunellae spica*, *Pseudobulbus cremastrae Seu pleiones*, *Radix bupleuri*, *licorice*, *Polygonum multiflorum*, *Crassostrea gigas* and *Cornu cervi*, and has been used for the treatment of HMG for several decades as a cipher prescription based on the clinical experience of many medical experts. Clinical researches showed that the total effective rate of LRK in the treatment of HMG was 88.0%. Moreover, LRK showed obvious superiority in improving the patients’ symptoms and abnormalities of gonadal hormone (Qian et al., 2007).

As a rapidly developing technology, metabolomics can discover the pathogenesis of diseases by detecting more than 1,000 molecules in various biological fluids, such as urine, saliva, and blood. This technology has been successfully applied to the diagnosis and identification of various diseases, such as coronary heart disease (Li et al., 2017c), early chronic kidney disease (Benito et al., 2018) and thyroid cancer (Navas-Carrillo et al., 2017). Metabolomics has also been widely applied in the field of TCM (Li et al., 2017b; Liu et al., 2017) and provides insight into the complex network mechanisms of HMG. In addition, network pharmacology has also been successfully applied to the study of TCM molecular mechanisms and can provide a deep understanding of the complex relationship between TCM components and diseases.

In this study, we elucidated the bioactive components, potential biomarkers and possible mechanisms of LRK in the treatment of HMG using metabolomics and network pharmacology.

## Materials and Methods

### Water Extract of LRK Preparation

*Curcumae radix, Prunellae spica, Pseudobulbus cremastrae Seu pleiones, Radix bupleuri, licorice, Polygonum multiflorum, Crassostrea gigas* and *Cornu cervi* were purchased from He yanling, Co., Ltd. (Beijing, China). The origin and quality of the 8 herbs were identified according to the Chinese Pharmacopeia (2015 Edition). Water extract from LRK was prepared by extracting the mixture of the 8 herbs (at a ratio of 12: 12: 9: 10: 30: 10: 9: 6, respectively) twice with water for 1 h. The extract was then decanted, filtered and dried under reduced pressure. The final ratio of powder to raw herb was 9.2%.

### Animals

Female Sprague-Dawley rats weighing 200 ± 5 g (license number: SCXK-(A) 2012-0004) were obtained from the laboratory animal center of Military Medical Science Academy of the PLA. All studies were performed in accordance with the Guiding Principles for the Care and Use of Laboratory Animals of China. The animals were housed in the same feeding environment (temperature: 25 ± 2°C; humidity: 50 ± 10%) and fed based on lighting conditions (12:12 h light: dark cycle) with a standard diet and water *ad libitum*. After acclimatization for 7 days, all animals were randomly divided into six groups (*n* = 6): a normal group (A), a model group (B), a tamoxifen-treated HMG group (C), a low dose LRK-treated HMG group (D), a medium dose LRK-treated HMG group (E) and a high dose LRK-treated HMG group (F). Rats in the B, C, D, E, and F groups were intramuscularly injected with estrogen (0.5 mg/kg/d) for 25 days, followed by progestogen (5 mg/kg/d) for another 5 days to induce the experimental HMG model (Wang et al., 2011). Rats in groups D, E, and F were given 0.6, 1.2 and 2.4 g/kg LRK orally for 30 days, respectively. Rats in groups A and B were treated with an equal volume of saline. Rats in group C were given oral tamoxifen (4 mg/kg/d) for 30 days. All animal studies were approved by the Ethical Committee of 302 Military Hospital of China.

### Sample Preparation

After the last treatment, all rats were sacrificed. Blood samples were collected and centrifuged at 3000 ×*g* for 10 min to obtain serum for mass spectrometry. The mammary samples were rapidly excised and fixed in 10% paraformaldehyde solution for histopathological analysis. Paraffin-embedded sections ranging from 4 to 5 μm in thickness were stained with haematoxylin-eosin (H & E). The stained sections were analyzed with a Nikon microscope (Nikon Instruments Corporation, Shanghai, China) and Image-Pro Plus 7200 software.

### Sample Handling

Briefly, 200 μL of serum samples and 600 μL of methanol were mixed uniformly and allowed to stand for 20 min at 4°C. The samples were centrifuged at 12,000 rpm at 4°C for 10 min to obtain the supernatant and then filtered through a syringe filter (0.22 μm) to obtain the sample for injection.

### Chromatography and Mass Spectrometry

Chromatography was carried out using an Agilent 1290 series UHPLC system. The sample injection volume was 4 μL, and all samples were detected at 4°C on a ZORBAX RRHD 300 SB-C18 column (2.1 mm × 100 mm, 1.8 μm; Agilent, United States). The mobile phases were composed of 0.1% formic acid in acetonitrile (solvent A) and 0.1% formic acid in water (solvent B), and the flow rate was 0.30 mL/min. The gradient was set as follows: the first minute: 95% A; 1.0 to 9.0 min: 95–60% A; 9.0 to 19.0 min: 60–10% A; 19.0 to 21.0 min: 10–0% A; and 21.0 to 25.0 min: 0% A. After injection of the 10 samples and the QC sample compounded with all samples, a blank was injected to guarantee the stability and repeatability of the UPLC-QTOF/MS systems.

Mass spectrometry was performed using an Agilent 6550 Q-TOF/MS instrument (Agilent Technologies, Santa Clara, CA, United States) with an electrospray ionization source (ESI) in both positive and negative ionization mode. The following electrospray source parameters were used: the electrospray capillary voltages were 3.0 kV (negative ionization mode) and 4.0 kV (positive ionization mode); the gas temperature was 200°C (negative ionization mode) and 225°C (positive ionization mode); the mass range ranged from m/z 80 to 1000; the gas flow was 11 L/min; the nebulizer was 35 pisg in negative ionization mode and 45 pisg in positive ionization mode; the sheath gas temperature was set to 350°C, and the sheath gas flow was 12 L/min; the nozzle voltage was 500 V in both negative and positive ionization modes.

### Data Extraction and Multivariate Analysis

MassHunter Profinder software (Agilent, California, United States) was used to extract sample data for peak detection and alignment. Full scan mode was applied in the mass range of 80–1000 m/z. The initial and final retention times were set for data collection. The resultant data matrices were normalized using MetaboAnalyst 3.0^1^ and then introduced to SIMCA-P 13.0 software (Umetrics, Umea, Sweden) for PCA and PLSDA analysis. PCA was used as an unsupervised pattern recognition approach to reduce the dimension of the UPLC-QTOF/MS data and disclose intrinsic clustering of samples. PLS-DA analysis was employed to maximize the differences in inter-class discrimination and minimize the differences in inter-class discrimination. The variables with VIP >1.5 and |p(corr)|≥0.58 in the PLS-DA analysis were further evaluated with an independent sample *t*-test.

### Biomarkers Identification and Pathway Enrichment Analysis

The significant variables (*p* < 0.05 in ANOVA) were selected as potential biomarkers for further pathway enrichment analysis. These biomarkers were identified by METLIN^2^, and the identified compound names were resubmitted to MetaboAnalyst 3.0 to analyse their signaling pathways.

### Identification of Drug Targets and Potential Metabolites and Network Construction

To systematically elucidate the complex relationships between potential metabolites and their associated targets, we conducted network analysis using network pharmacology. The ingredients with an oral bioavailability (OB) ≥30% and a drug-likeness (DL) >0.18 from *Radix bupleuri*, *licorice*, *Pseudobulbus cremastrae Seu pleiones*, *Prunellae spica*, *Polygonum multiflorum* and *Curcumae radix* were put into the TCMSP database^3^, and their corresponding chemical components were also collected from the same database (Huang et al., 2017). The ingredients and corresponding targets of *Cornu cervi* and *Crassostrea gigas* were retrieved from the BATMAN-TCM database^4^. The protein targets of potential metabolites were collected from the MBROLE 2.0 database^5^ (Lopez-Ibanez et al., 2016). Different protein ID types were converted to UniProt IDs. Then, the “metabolite-target-chemical components” interactive network was established by using protein–protein interaction (PPI) information. Cytoscape 2.8.3 (National Institute of General Medical Sciences, United States) was applied to visualize and analyse the network.

### Immunohistochemistry

To evaluate the effect of LRK on the expression of PKCα in breast tissue of HMG rats, immunohistochemistry was performed as described previously (Chen et al., 2014). Breast tissue slides were incubated with anti- PKCα (Abcam, Cambridge, MA, United States) for 12 h at 4°C. After treatment with HRP-conjugated goat anti-mouse IgG and 50 μL of streptavidin-peroxidase solutions for 30 min at RT, the sections were stained with DAB and counterstained with hematoxylin. The positive areas showed the color of brown yellow.

### Statistical Analysis

Data were expressed as the mean ± SE and analyzed with the SPSS 13.0 software program (Chicago, United States). The differences between the group means were calculated by ANOVA. *p* < 0.05 was considered statistically significant, and *p* < 0.01 was considered highly significant. The results of the three-dimensional matrix containing the peak index, sample name, and peak area were introduced into SIMCA-P 13.0 software for pattern recognition analysis of PCA and PLS-DA.

## Results

### Therapeutic Effects of LRK on HMG

**FIGURE 1:**
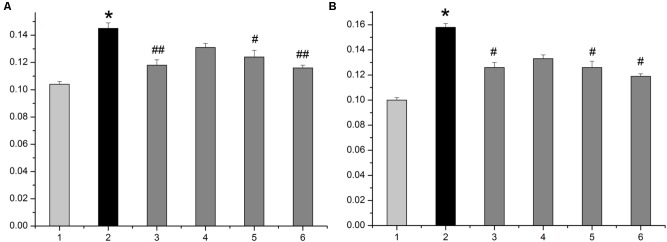
The average diameter and height of nipples in the HMG rats. **(A)** The average diameter of nipples. **(B)** The average height of nipples. 1: normal group. 2: model group. 3: tamoxifen-treated HMG group (4 mg/kg). 4: low-dose LRK-treated HMG group (0.6 g/kg). 5: medium-dose LRK-treated HMG group (1.2 g/kg). 6: high-dose LRK-treated HMG group (2.4 g/kg). Data are expressed as the mean ± SE (*n* = 6). ^∗^*p* < 0.01 compared with the normal group; ^##^*p* < 0.01, ^#^*p* < 0.05 compared with the model group.

**FIGURE 2:**
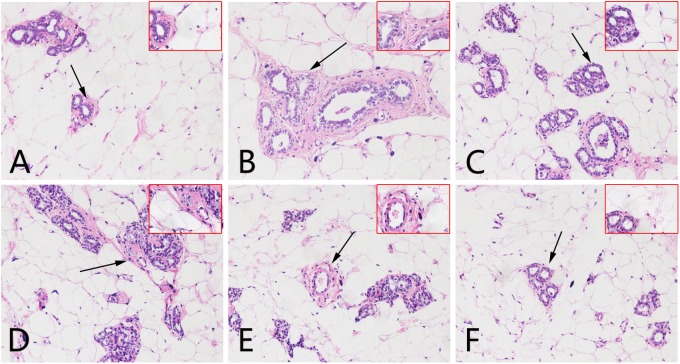
Effects of LRK on the histopathology of mammary tissues using haematoxylin and eosin staining (200× and 400×). **(A)** Normal group. **(B)** Model group. **(C)** Tamoxifen-treated HMG group (4 mg/kg). **(D)** Low-dose LRK-treated HMG group (0.6 g/kg). **(E)** Medium-dose LRK-treated HMG group (1.2 g/kg). **(F)** High dose LRK-treated HMG group (2.4 g/kg). The magnified areas (×400) are marked with a black arrow in the pathological tissue figures (200×).

The average diameter and height of nipples (Left 2 and Right 2) were measured as indices of HMG. As shown in **Figure 1**, rats given estrogen and progesterone displayed remarkable increases in the average nipple diameter and height. The administration of LRK (2.4 and 1.2 g/kg) markedly decreased the average nipple diameter and height, respectively. Pathological studies provided direct evidence of protective effects of LRK on HMG. In **Figure 2**, the mammary tissues of the rats in the normal group exhibited normal morphological structures with no abnormal features. By contrast, the mammary tissues of the rats in the model group displayed more acinus and lobules, expanded mammary lumens, and hyperplastic ducts. Conversely, treatment with LRK (2.4 and 1.2 g/kg) ameliorated these morphological changes. These results preliminarily verified that 2.4 and 1.2 g/kg LRK significantly protected the mammary tissue in the rats with HMG induced by estrogen and progesterone.

### Multivariate Statistical Analysis

**FIGURE 3:**
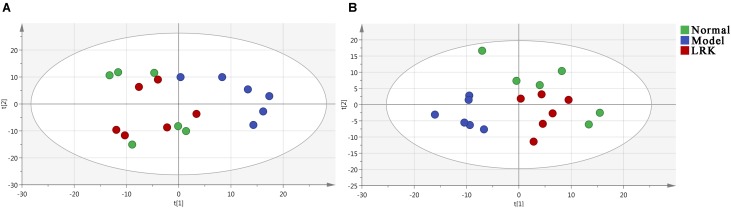
Principal component analysis (PCA) score plot of the control, model and LRK (2.4 g/kg) groups. **(A)** ESI+ model. **(B)** ESI- model.

Principal component analysis (PCA) and orthogonal partial least squares-discriminant analysis (OPLS-DA) are pattern recognition approaches frequently used to classify metabolic phenotypes and identify different metabolites to evaluate variation among complex data sets. Score plots revealed a direct image of observational clusters. A distinguished classification between the clustering of the normal and model groups and the normal and LRK groups was observed in both the positive (**Figure 3A**) and negative modes (**Figure 3B**). Further multivariate analysis was needed to explore which metabolites caused these differences.

**FIGURE 4:**
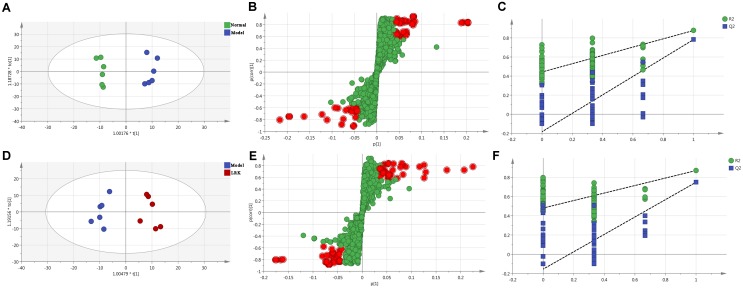
The OPLS-DA score plots, S-plots and 100-permutation test generated from the OPLS-DA data of the normal, model and LRK groups in ESI+ mode. OPLS-DA score plots were the pair-wise comparisons between the normal and model groups **(A)** as well as between the model and LRK groups **(D)**. S-plots of the OPLS-DA model for the normal and model groups **(B)** as well as for the model and LRK groups **(E)**. The 100-permutation test of the OPLS-DA model was for the normal and model groups **(C)** as well as for the model and LRK groups **(F)**.

Orthogonal partial least squares-discriminant analysis was used to identify potential biomarkers that were significantly changed between the normal, model and LRK groups. The parameters R^2^X (cum), R^2^Y (cum), and Q^2^ (cum) were used to provide an estimate of how well the model fit the data. We further determined whether the metabolite fingerprints in the serum differed between the normal, model, and LRK groups by constructing an OPLS-DA model. A distinguished classification between the clustering of the normal, model, and LRK groups was observed in both the positive (**Figure 4**) and negative models (Supplementary Figure S1), suggesting a significant serum biochemical perturbation in the model and LRK groups. The R^2^X (cum), R^2^Y (cum), and Q^2^ (cum) of OPLS-DA in our positive model were 0.506, 0.936, 0.79, respectively, using the data from the control and model groups and 0.304, 0.87, 0.748, respectively, using the data from the model and LRK groups. The R^2^X (cum), R^2^Y (cum), and Q^2^ (cum) of OPLS-DA in our negative model were 0.261, 0.908, 0.768, respectively, using the data from the control and model groups and 0.233, 0.97, 0.806, respectively, using the data from the model and LRK groups. The corresponding parameter results indicated that models were of good quality and provided accurate predictions. Variables further away from the origin in the corresponding S-plots were thought to contribute more significantly were more thus responsible for the separation between the normal and model groups as well as the model and LRK groups in both the positive (**Figures 4B,E**) and negative ionization models (Supplementary Figure S1). These variables may therefore be regarded as potential biomarkers. Permutation tests with 100 iterations were performed to validate the model. These tests compared the goodness of fit of the original model with the goodness of fit of randomly permuted models. As shown in **Figures 4C,F**, the validation plots indicated that the original models were valid.

### Identification of Potential Metabolites in HMG Treatment

Among the 1986 signals detected in the control, model and 2.4 g/kg LRK groups, variables that significantly contributed to the clustering and discrimination were identified according to a threshold of VIP ≥1.5 and |p(corr)|≥ 0.58. Those thresholds were obtained after OPLS-DA processing these variables. According to the VIP and |p(corr)|, 366 variables were selected from the control, model and 2.4 g/kg LRK groups as the candidates for fold-changes and ANOVA analyses. The candidates that significantly differed among the groups with fold change exceeding two were identified as candidate biomarkers for METLIN and Metaboanalyst identification. Ten potential biomarkers were summarized in **Table 1** with their corresponding formula, retention time, m/z, and differences by group.

**Table 1:** Identified metabolites of the serum from different groups.

No	Metabolites	Formula	R.T. (min)	Mass (m/z)	Ratio changes (significance)	
					Control/Model	LRK/Model
1	Sulfolithocholylglycine	C_26_H_42_NO_7_S	7.36	536.2628	0.40ˆ**	0.45ˆ##
2	Leukotriene B4	C_20_H_32_O_4_	6.57	339.2557	0.23ˆ**	0.16ˆ##
3	Phosphatidylcholine	C_10_H_18_NO_8_PR_2_	6.9	792.5771	1.36ˆ**	1.07ˆ#
4	Ceramide	C_19_H_36_NO_3_R	6.57	678.6891	3.10ˆ**	2.98ˆ##
5	Prostaglandin E2	C_20_H_32_O_5_	10.99	375.2233	3.50ˆ**	2.72ˆ##
6	3-Hydroxypicolinic acid	C_6_H_5_NO_3_	1.25	173.9994	0.68ˆ**	0.67ˆ##
7	5-Amino-4-imidazole carboxylate	C_4_H_5_N_3_O_2_	1.06	162.006	3.88ˆ**	3.04ˆ##
8	Phosphatidylserine	C_8_H_12_NO_10_PR_2_	6.96	854.5852	6.43ˆ**	3.23ˆ##
9	Coenzyme Q9	C_54_H_82_O_4_	6.95	829.5664	0.14ˆ**	0.58ˆ##
10	Gamma-glutamyl-L-putrescine	C_9_H_19_N_3_O_3_	4.73	216.1361	0.20ˆ**	0.51ˆ##

*^∗∗^P < 0.01 compared with normal group; ^##^P < 0.01, ^#^P < 0.05 compared with model group.*

### Pathway Analysis of HMG Treatment

**FIGURE 5:**
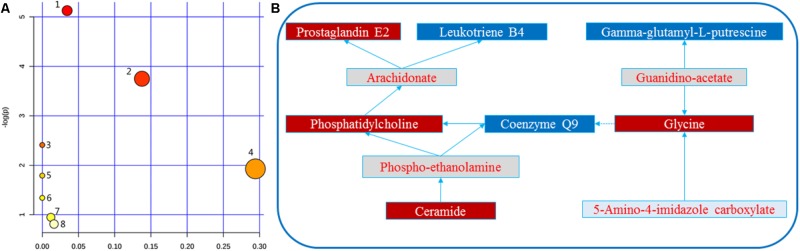
**(A)** Metabolomic Pathway construction of the metabolic pathways involved in the effects of LRK on HMG. **(B)** Signaling networks associated with the differentially expressed metabolic pathways. 1: Arachidonic acid metabolism. 2: Glycerophospholipid metabolism. 3: Linoleic acid metabolism. 4: Sphingolipid metabolism. 5: alpha-linolenic acid metabolism. 6: Glycine, serine and threonine metabolism. 7: Arginine and proline metabolism. 8: Purine metabolism. The red solid box represents the peak area of the LRK/model >1. The blue solid box represents the peak area of LRK/model < 1.

Herein, 10 potential metabolites were expressed at significant levels. Pathway analysis was performed in detail using Metaboanalyst. HMG-associated metabolites were responsible for metabolizing arachidonic acid, glycerophospholipid, linoleic acid, sphingolipid, alpha-linolenic acid, glycine, serine, threonine, arginine, proline, and purine (**Table 2** and **Figure 5**). The top three metabolic pathways were arachidonic acid, glycerophospholipid and linoleic acid.

**Table 2:** Results of integrating enrichment analysis of biomarkers with MetaboAnalyst 3.0.

No	Pathway Name	Match Status	*p*	-log(p)	Impact
1	Arachidonic acid metabolism	3/62	0.0059386	0.0059386	0.0059386
2	Glycerophospholipid metabolism	2/39	0.023519	0.023519	0.023519
3	Linoleic acid metabolism	1/15	0.048758	0.089758	0.089758
4	Sphingolipid metabolism	1/25	0.14536	0.14536	0.14536
5	alpha-Linolenic acid metabolism	1/29	0.16669	0.16669	0.16669
6	Glycine, serine and threonine metabolism	1/48	0.26143	0.26143	0.26143
7	Arginine and proline metabolism	1/77	0.38685	0.94973	0.01185
8	Purine metabolism	1/92	0.44363	0.81278	0.0162

### “Potential Metabolite-Target-Component” Interactive Network and Analysis

**FIGURE 6:**
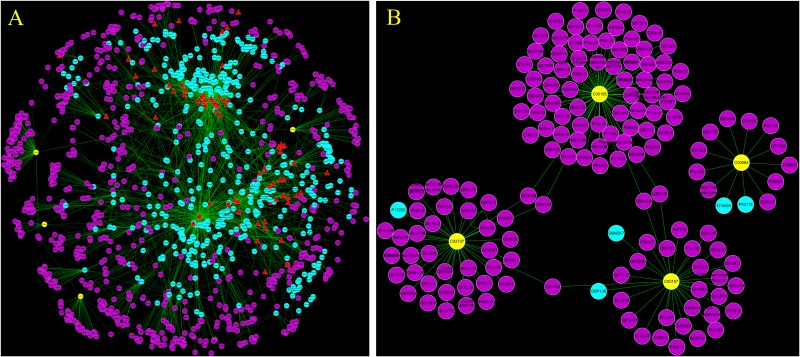
The “potential metabolite-target-component” interactive network with all target information **(A)** and key target information **(B)** participating in the treatment of HMG by LRK. The red triangles represent active chemical constituents of LRK. The blue dots represent the protein targets of drugs. The yellow dots represent potential metabolites. The purple dots represent the targets associated with potential metabolites.

**FIGURE 7:**
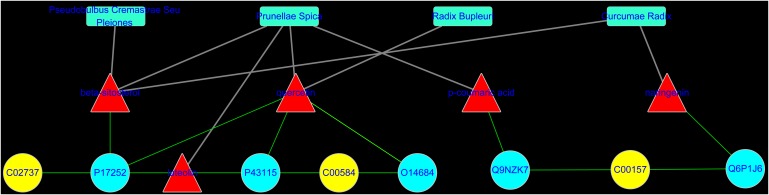
The “potential metabolite-target-component-herb” interactive network participating in the treatment of HMG by LRK. The red triangles represent active chemical constituents of LRK. The blue dots represent the protein targets of herbs. The yellow dots represent the potential metabolites. The blue rectangles represent herbs.

To reveal the proteins targets and chemical components of LRK regulation, the “potential metabolite-target-component” interactive network was structured by combing the drug targets and the targets associated with potential metabolites. As shown in **Figure 6**, 445 drug targets, 170 targets associated with potential metabolites, and 3 potential metabolites, including phosphatidylserine (C02737), prostaglandin E2 (C00584) and phosphatidylcholine (C00157), participated in the “potential metabolite-target-component” interactive network (**Figure 6A**). Five drug targets, including *protein kinase C alpha type* (P17252)*, prostaglandin E synthase* (O14684)*, prostaglandin E2 receptor EP3 subtype* (P43115)*, group IIE secretory phospholipase A2* (Q9NZK7) and *phospholipase B1* (Q6P1J6), directly regulated the 3 potential metabolites (**Figure 6B**). The 5 drug targets were directly regulated by multiple chemical components, including *beta-sitosterol, quercetin, p-coumaric acid* and *naringenin*, which were collected from 4 herbs, including *Curcumae radix, Prunellae spica, Radix bupleuri* and *Pseudobulbus cremastrae Seu pleiones* in LRK (**Figure 7**).

### The Effect of LRK on the Expression of PKCα in Breast Tissue of HMG Rats

**FIGURE 8:**
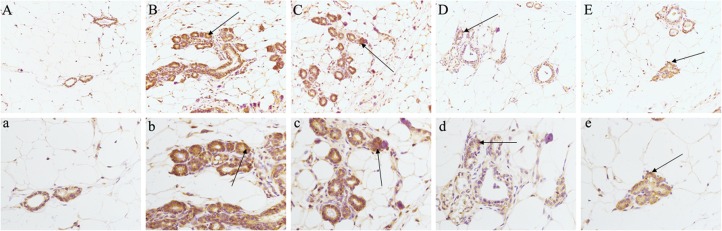
Effects of LRK on the expression of PKCα in breast tissue of HMG rats using immunohistochemistry (200× and 400×). **(A)** (200**×**) and **a** (400**×**) : normal group. **(B)** (200**×**) and **b** (400**×**): model group. **(C)** (200**×**) and **c** (400**×**): low-dose LRK-treated HMG group (0.6 g/kg). **(D)** (200**×**) and **d** (400**×**): medium-dose LRK-treated HMG group (1.2 g/kg). **(E)** (200**×**) and **e** (400**×**): high dose LRK-treated HMG group (2.4 g/kg).

Analysis of the network pharmacology prediction results showed that the greatest number of chemical components directly connected to PKCα, suggesting that PKCα may play a more important role in the treatment of HMG by LRK. Therefore, to verify the authenticity of the network pharmacology prediction results, we evaluated the expression of PKCα in breast tissue by immunohistochemistry. The results indicated the expression of PKCα was significantly increased in the breast tissues of rats in the model group, while the different doses of LRK significantly reduced the expression of PKCα (**Figure 8**).

## Discussion

The morbidity rate of HMG is increasing annually and continues to affect younger women (Zhang et al., 2008). HMG is classified in the “Rupi” category according to TCM theory (Fan et al., 2013). While network pharmacology and metabonomics have been applied to TCM research (Su et al., 2017; Wang et al., 2017), few studies have attempted to combine these two approaches. Metabolomics can analyze and detect small molecules in bodily fluids to determine which compounds have significant abnormalities and to uncover the mechanism of disease development. Network pharmacology can analyze the interaction of macromolecule targets corresponding to chemical components and diseases and reveal the molecular mechanisms of drug treatment. By combining metabolomics and network pharmacology, we were able to gain a deeper understanding of the molecular mechanisms underlying TCM treatment.

Li-Ru-Kang is made up of 8 kinds of herbs. It has been effective in treating HMG for decades. In this study, we combined metabonomics and network pharmacology analyses to evaluate the efficacy, active components and possible molecular mechanisms of LRK in the treatment of HMG. LRK significantly improved the morphological characteristics of nipples and reduced the pathological state of HMG in rats. Furthermore, we described the metabolomic feature profile and metabolite interaction network of LRK in treating HMG. LRK exhibited protective effects against HMG by reversing potential metabolites to normal levels. Ten metabolites were significantly regulated by LRK. Seven of these metabolites, including prostaglandin E2, leukotriene B4, phosphatidylcholine, coenzyme Q9, ceramide, gamma-glutamyl-L-putrescine and 5-amino-4-imidazole carboxylate, could interact in different ways. The results indicated that the occurrence and development of HMG were caused by changes in many aspects of physiologically and pathologically related molecules and that most of the changes in the body’s molecules were interrelated. LRK regulated these 10 biomarkers to normalize their expression levels, indicating that LRK could be used to treat HMG via multiple pathways and multiple targets.

To obtain a deeper understanding of the LRK mechanism for treating HMG and the correlation between LRK chemical components and metabolites, we further combined the network pharmacology method to establish the “potential metabolite-target-component” interactive network. The results showed that four chemical components, *beta-sitosterol, quercetin, p-coumaric acid* and *naringenin*, which come from *Curcumae radix, Prunellae spica, Radix bupleuri* and *Pseudobulbus cremastrae Seu pleiones*, acted directly on multiple targets that are directly related to metabolites, including *prostaglandin E synthase, protein kinase C alpha type, prostaglandin E2 receptor EP3 subtype, group IIE secretory phospholipase A2* and *phospholipase B1*. The results suggested that multiple components in LRK played an important role in regulating the endogenous physical disturbance of the body, thus indicating the advantages of LRK in the clinical treatment of HMG. By investigating the pathogenesis of HMG, we found that inflammation and oxidative stress mechanisms played a key role in the development of HMG (Holloway et al., 2012). At the same time, some drugs act on HMG by exerting anti-inflammatory and antioxidant pharmacological effects (Das Gupta et al., 2015; Huang et al., 2017). Studies have shown that beta-sitosterol (Villasenor et al., 2002; Tan et al., 2008), quercetin (Kamaraj et al., 2007), p-coumaric acid (Ilavenil et al., 2016) and naringenin (Cavia-Saiz et al., 2010; Chen et al., 2013) in LRK have anti-inflammatory and antioxidant pharmacological activities. The network pharmacology results showed that PKCα was the target directly connected to the four chemical components. This target is also the most closely related target to inflammation and oxidative stress (Kopach et al., 2013; Andersson et al., 2016). Therefore, to verify the accuracy of the network pharmacology prediction results, we further examined the expression of PKCα in the breast tissues of HMG rats. The expression of PKCα was increased significantly in HMG rats, and LRK significantly reduced PKCα expression. These results suggested that the network pharmacology prediction results were credible. This experiment also demonstrated the significance of PKCα in the pathogenesis of HMG and LRK treatment.

This study systematically explored the molecular mechanism of LRK for the treatment of HMG by combining metabonomics and network pharmacology analyses. In addition, this innovative research method can provide new ideas for research on the molecular mechanisms of TCM in the treatment of complex diseases.

## Author Contributions

SW and LQ performed the experiments, analyzed the data, and wrote the manuscript. MN, HHL, YY, YW, LZ, XZ, and HTL collected and prepared samples. RW, KL, and YZ performed the analyses.

## Conflict of Interest Statement

The authors declare that the research was conducted in the absence of any commercial or financial relationships that could be construed as a potential conflict of interest.

## Footnotes

1 http://www.metaboanalyst.ca/

2 http://metlin.scripps.edu/

3 http://lsp.nwu.edu.cn/tcmsp.php

4 http://bionet.ncpsb.org/batman-tcm/

5 http://csbg.cnb.csic.es/mbrole2/
