# Supplementary material for: The Modulatory Properties of Li-Ru-Kang Treatment on Hyperplasia of Mammary Glands Using an Integrated Approach
Source: Front Pharmacol. 2018 Jun 19;9:651. doi: 10.3389/fphar.2018.00651 (PMC6018463; doi:10.3389/fphar.2018.00651)
Supplement: Supplementary file 1 [file Data_Sheet_1.DOCX]

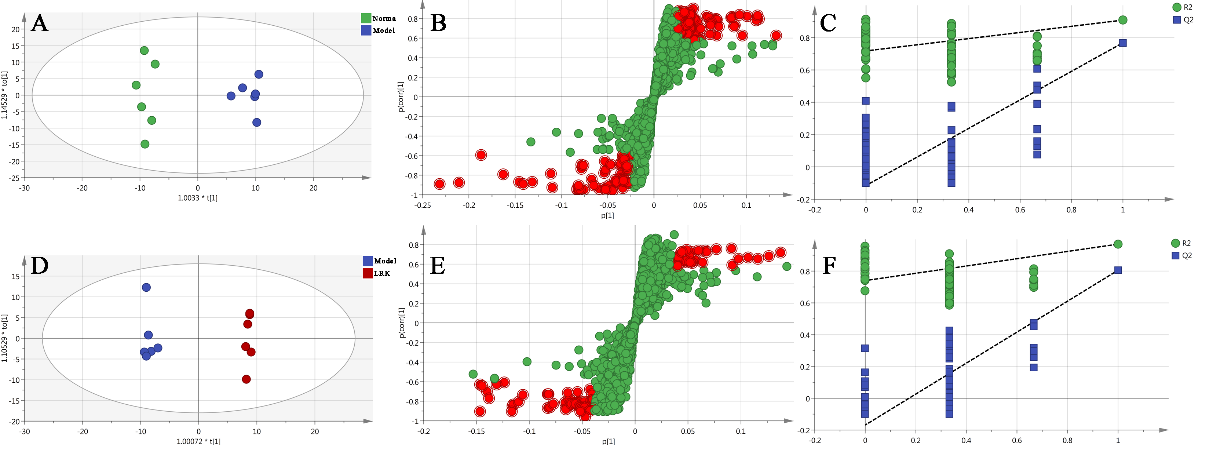


**Figure S1** The OPLS-DA score plots, S-plots and 100-permutation test generated from the OPLS-DA data of the normal, model and LRK groups in ESI- mode. OPLS-DA score plots were the pair-wise comparisons between the normal and model groups (A) as well as between the model and LRK groups (D); S-plots of the OPLS-DA model were for the normal and model groups (B) as well as for the model and LRK groups (E); 100-permutation test of OPLS-DA model was for the normal and model groups (C) as well as for the model and LRK groups (F).
